# Probenecid Inhibits the Human Bitter Taste Receptor TAS2R16 and Suppresses Bitter Perception of Salicin

**DOI:** 10.1371/journal.pone.0020123

**Published:** 2011-05-24

**Authors:** Tiffani A. Greene, Suzanne Alarcon, Anu Thomas, Eli Berdougo, Benjamin J. Doranz, Paul A. S. Breslin, Joseph B. Rucker

**Affiliations:** 1 Integral Molecular, Inc., Philadelphia, Pennsylvania, United States of America; 2 Department of Nutritional Sciences, School of Environmental and Biological Sciences, Rutgers University, New Brunswick, New Jersey, United States of America; 3 Monell Chemical Senses Center, Philadelphia, Pennsylvania, United States of America; Duke University, United States of America

## Abstract

Bitter taste stimuli are detected by a diverse family of G protein-coupled receptors (GPCRs) expressed in gustatory cells. Each bitter taste receptor (TAS2R) responds to an array of compounds, many of which are toxic and can be found in nature. For example, human TAS2R16 (hTAS2R16) responds to β-glucosides such as salicin, and hTAS2R38 responds to thiourea-containing molecules such as glucosinolates and phenylthiocarbamide (PTC). While many substances are known to activate TAS2Rs, only one inhibitor that specifically blocks bitter receptor activation has been described. Here, we describe a new inhibitor of bitter taste receptors, *p*-(dipropylsulfamoyl)benzoic acid (probenecid), that acts on a subset of TAS2Rs and inhibits through a novel, allosteric mechanism of action. Probenecid is an FDA-approved inhibitor of the Multidrug Resistance Protein 1 (MRP1) transporter and is clinically used to treat gout in humans. Probenecid is also commonly used to enhance cellular signals in GPCR calcium mobilization assays. We show that probenecid specifically inhibits the cellular response mediated by the bitter taste receptor hTAS2R16 and provide molecular and pharmacological evidence for direct interaction with this GPCR using a non-competitive (allosteric) mechanism. Through a comprehensive analysis of hTAS2R16 point mutants, we define amino acid residues involved in the probenecid interaction that result in decreased sensitivity to probenecid while maintaining normal responses to salicin. Probenecid inhibits hTAS2R16, hTAS2R38, and hTAS2R43, but does not inhibit the bitter receptor hTAS2R31 or non-TAS2R GPCRs. Additionally, structurally unrelated MRP1 inhibitors, such as indomethacin, fail to inhibit hTAS2R16 function. Finally, we demonstrate that the inhibitory activity of probenecid in cellular experiments translates to inhibition of bitter taste perception of salicin in humans. This work identifies probenecid as a pharmacological tool for understanding the cell biology of bitter taste and as a lead for the development of broad specificity bitter blockers to improve nutrition and medical compliance.

## Introduction

As the primary mechanism by which animals detect and evaluate nutrients within foods and avoid ingesting toxins, the sense of taste has a significant impact on food selection, nutrition, and health. For example, the sense of taste in individuals is reported to correlate with a variety of habits including dietary preference [1], alcohol intake [2], smoking [3], and patient compliance with medical regimens [4]. It is therefore highly desirable to manipulate bitter taste perception and bitter taste receptors so that beneficial food products and medicines may be rendered more palatable. Recently, bitter taste receptors have been implicated in several aspects of respiratory and gastrointestinal function, hinting at a broader biological role for this receptor family [5], [6], [7], [8], [9], [10], [11]. Therefore, bitter taste receptor modulation may also represent a new approach for understanding the function of bitter taste receptors in non-gustatory cells of airway epithelia, smooth muscle, and the intestine.

Bitter substances are recognized by, and bind to, a family of taste receptors (TAS2Rs) that are expressed in bitter taste cells, and initiate signaling via activation of intracellular heterotrimeric G proteins [12], [13], [14], [15], [16], [17]. TAS2Rs are phylogenetically distinct from the canonical rhodopsin (class A) receptor family and more closely related to the *frizzled/smoothened* family of GPCRs [18]. There are at least 25 human full-length TAS2Rs, clustered on 3 human chromosomes, which are highly divergent in sequence, sharing between 30–70% amino acid homology [18]. Additionally, there are a large number of TAS2R pseudogenes (over 30% of the human TAS2R repertoire), and there are more than 80 single nucleotide polymorphisms (SNPs) among individual TAS2R genes [19], [20], several of which result in variation in the range and intensity of various human bitter taste perceptions [21], [22], [23], [24]. Unlike most GPCRs, TAS2Rs recognize a diverse variety of chemical moieties. While many bitter taste receptors remain poorly characterized, the ligand specificity of several TAS2Rs has been explored in detail. These include hTAS2R16, which responds to β-glucosides such as salicin [25], hTAS2R38, which responds to thiourea-containing molecules such as the drugs phenylthiocarbamide (PTC) and 6-propyl-2-thiouracil (PROP) [21], and hTAS2R43 and hTAS2R31 (formerly known as hTAS2R44), a closely related pair of receptors that transduce the signal for the bitter taste of saccharin [23], [26]. Despite the diversity of chemicals recognized by TAS2Rs and the continued interest in developing bitter blockers to mask the bitter taste of drugs and certain foods, only a single synthetic inhibitor against this class of GPCRs has been described to date [27]. The identification of additional compounds that inhibit TAS2Rs may help our understanding of the broader biological relevance of this class of receptors, particularly if they utilize diverse mechanisms of inhibition.

Probenecid (*p*-(dipropylsulfamoyl)benzoic acid) is an FDA-approved inhibitor of the organic anion transporter Multidrug Resistance Protein 1 (MRP1) and other organic anion transporters [28], [29]. Clinically, probenecid is used as a treatment for gout in humans [30], acting as a uricosuric agent, and is also co-administered with antibiotics and other chemotherapeutic agents to improve their efficacy by reducing their excretion. Within the laboratory, probenecid is commonly used to prevent the efflux of calcium-sensitive fluorescent dyes during studies of cellular calcium mobilization [31]. As such, many protocols for conducting GPCR calcium influx assays recommend *including* probenecid to facilitate dye loading. During the course of our studies of bitter taste receptor signaling, we unexpectedly discovered that probenecid *inhibited* the activation of the bitter taste receptor hTAS2R16 in response to its cognate ligand salicin. This activity occurred rapidly and was independent of probenecid's activity as a transport inhibitor, suggesting that probenecid interacts with the receptor rather than modulating downstream signaling processes. Consistent with its rapid inhibition, hTAS2R16 point mutations can suppress probenecid inhibition, suggesting a direct interaction with hTAS2R16 and an allosteric inhibitory mechanism in which the salicin and probenecid binding sites are distinct. Inhibition by probenecid was also observed for additional TAS2R receptors, including hTAS2R38 and hTAS2R43, but not for hTAS2R31 or for other non-gustatory GPCRs tested. In human perceptual studies, probenecid suppressed the bitter taste perception of salicin, demonstrating a correlation between the *in vitro* findings of probenecid inhibition and human bitter taste phenotype. The discovery of probenecid as an inhibitor of bitter taste receptors and human bitter perception offers insight into a molecular mechanism for designing modulators of human taste perception for improved food selection, nutrition, and health.

## Results

### Probenecid is an inhibitor of the hTAS2R16, hTAS2R38, and hTAS2R43 bitter taste receptors

**Figure 1:**
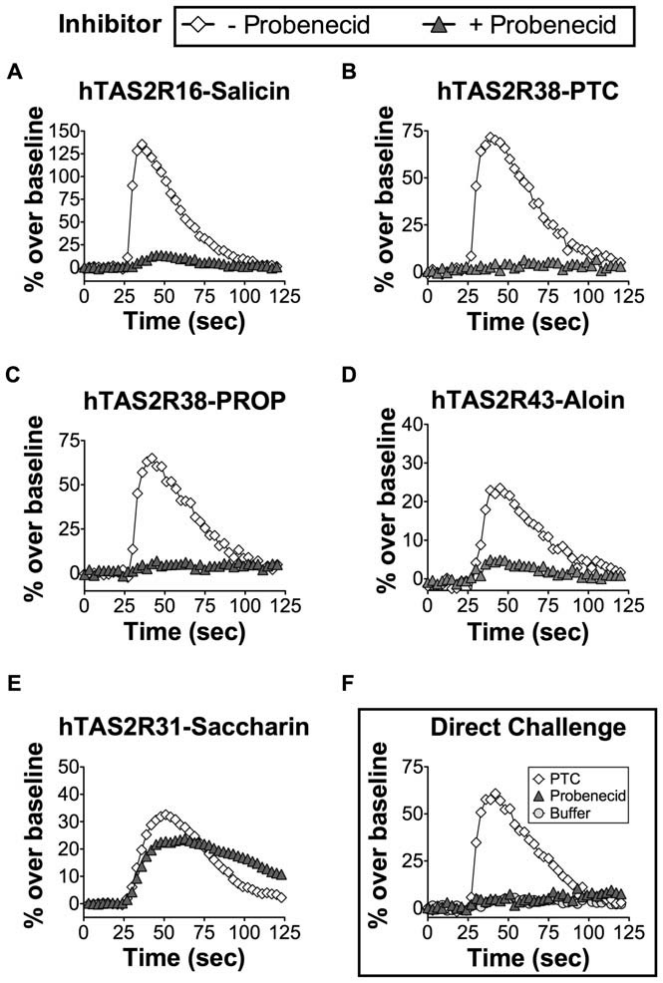
Inhibition of hTAS2R16, hTAS2R38, and hTAS2R43 by probenecid. HEK-293T cells were transiently transfected with Gα16gust44 and the indicated TAS2R receptors in a 384-well microplate. 22 hours post-transfection, calcium influx was measured in cells challenged with the indicated ligands in the presence (closed triangles) or absence (open diamonds) of probenecid (1 mM; 1 hour pre-incubation). Probenecid treatment completely attenuated (A) salicin-dependent (3 mM) calcium influx by the hTAS2R16 receptor and (B) PTC- (100 µM) and (C) PROP-dependent (30 µM) calcium influx by the hTAS2R38 receptor. (D) Probenecid treatment similarly attenuated aloin-induced (3 mM) hTAS2R43 signaling. (E) Probenecid treatment did not inhibit saccharin induced signaling of hTAS2R31. (F) hTAS2R38 transfected cells challenged with probenecid or buffer alone (1 mM) did not result in calcium influx, but do flux with the PTC control.

In order to study the cellular and molecular mechanisms of human bitter taste perception, we used an *in vitro* calcium flux assay in HEK-293T cells that monitors human bitter taste receptor activation and inhibition. The addition of salicin (3 mM) to HEK-293T cells transiently expressing hTAS2R16 and Gα16gust44 induces an increase in intracellular calcium levels that is measured using a Ca^2+^-activated fluorescent dye (Figure 1A). Probenecid is commonly used to improve the cellular uptake of various fluorescent dyes into cells and is typically recommended for improving the sensitivity of GPCR calcium flux assays [31]. It was therefore surprising that, upon a one hour pre-incubation with 1 mM probenecid (without washout), agonist responses of hTAS2R16 were attenuated to near baseline levels (Figure 1A).

**Figure 2:**
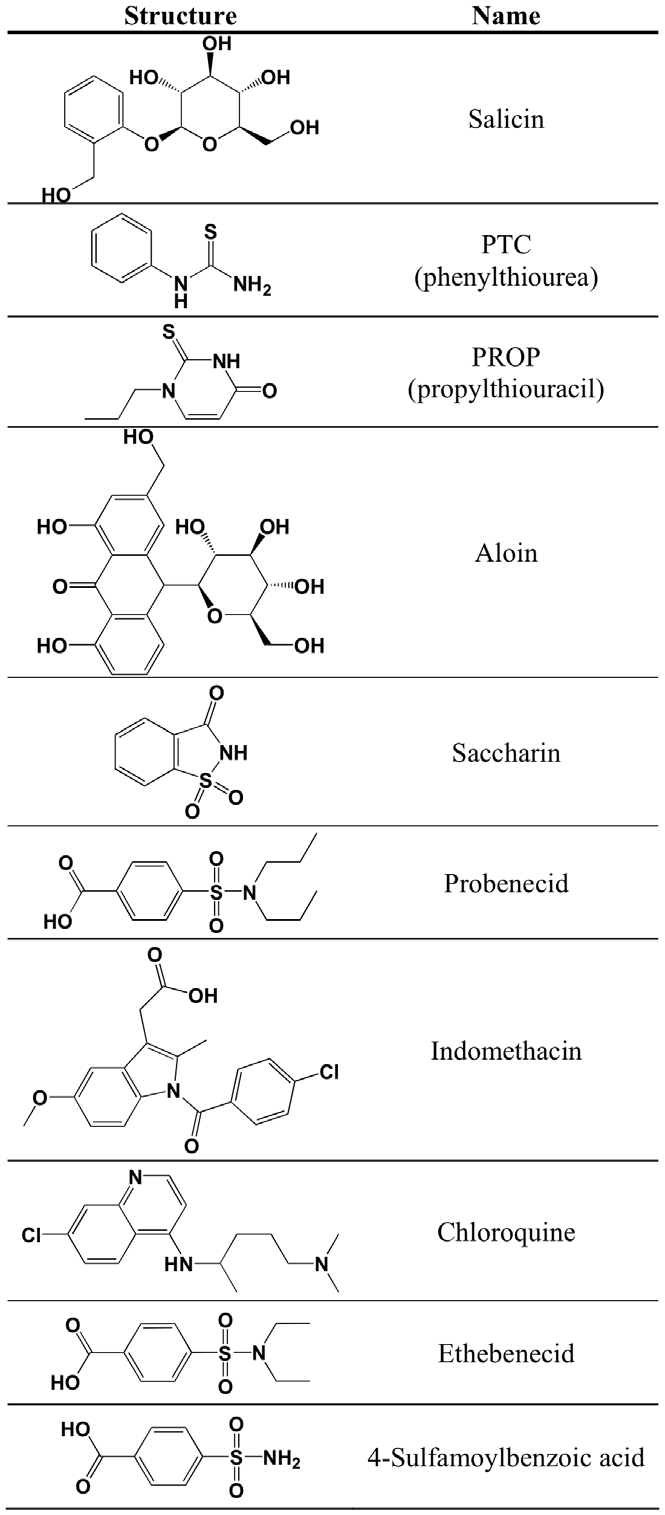
Structures of TAS2R agonists, probenecid, MRP1 inhibitors, and probenecid analogs used.

Using the calcium flux assay, we tested for probenecid inhibition of other TAS2Rs. Similar to hTAS2R16, pre-incubation with probenecid resulted in inhibition of hTAS2R38 activation by both PTC and by PROP (Figure 1B and 1C), two different ligands of hTAS2R38. hTAS2R43 and hTAS2R31 (formerly known as hTAS2R44), two de-orphanized TAS2R receptors that share 25% and 24% amino acid sequence identity with hTAS2R16 respectively, were also tested. Aloin induced an increase in intracellular calcium in HEK-293T cells expressing hTAS2R43, and this signal was almost completely inhibited by a 1 hour pre-incubation with 1 mM probenecid (Figure 1D). In contrast, the saccharin-induced calcium flux in cells expressing hTAS2R31 was not inhibited by probenecid pre-treatment (Figure 1E). Because the ligands used for testing each of these TAS2Rs represent diverse structures (Figure 2), it is unlikely that probenecid is acting on the ligands themselves. Addition of 1 mM probenecid or buffer directly to transfected cells did not by itself result in any change in intracellular calcium (Figure 1F). Our data suggest that probenecid acts specifically against a subset of TAS2Rs that include hTAS2R16, hTAS2R38, and hTAS2R43.

**Figure 3:**
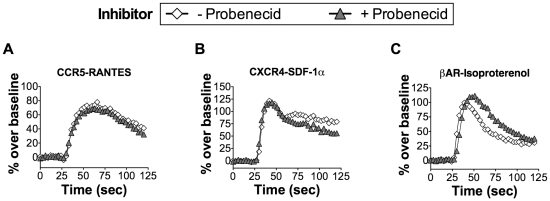
Non-Gustatory GPCRs are not inhibited by probenecid. HEK-293T cells were transiently transfected with Gα16gust44 and the indicated GPCR receptors. In the case of the endogenously expressed βAR receptor, only Gα16gust44 was transfected. 22 hours post-transfection, calcium influx was measured for cells that were challenged with the indicated ligands in the presence (closed triangles) or absence (open diamonds) of probenecid (1 mM; 1 hour pre-incubation). Probenecid treatment did not attenuate calcium influx upon challenge of (A) CCR5 with 10 nM RANTES, (B) CXCR4 with 10 nM SDF-1α, or (C) βAR with 10 µM isoproterenol.

We next examined whether probenecid could inhibit the cellular activation of the non-gustatory GPCRs CXCR4 and CCR5 when expressed in HEK-293T cells. The human chemokine receptors CXCR4 and CCR5 are members of the rhodopsin family of GPCRs, which are unrelated to the TAS2Rs and are involved in inflammation, autoimmune disease, and viral infection [32], [33]. We observed no effect on RANTES-mediated calcium mobilization by CCR5 and SDF-1α-mediated calcium mobilization by CXCR4 upon pre-incubation with probenecid (Figure 3A and 3B). We also tested the effect of probenecid on the activity of the β2-adrenergic receptor (βAR), which is endogenously expressed on HEK-293T cells [34]. Stimulation of endogenous βAR co-expressed with Gα16gust44 resulted in an increase in intracellular calcium upon stimulation with a cognate adrenergic ligand (isoproterenol) that was not inhibited by a 1 hr pre-incubation with probenecid (Figure 3C). Since βAR mobilizes calcium in HEK-293T cells only in the presence of transfected Gα16gust44, the inhibitory activity of probenecid is not the result of action through Gα16gust44, which would have led to inhibition of calcium influx. Probenecid also inhibited calcium mobilization of hTAS2R38 in the canine cell line Cf2Th, suggesting that the observed inhibition is not cell line specific (data not shown). While probenecid inhibition of the G protein βγ subunits cannot be directly ruled out with the experiments conducted here, it is unlikely since no effect on calcium mobilization was observed with other GPCRs tested and since probenecid is commonly used to enhance the Ca^2+^ flux of diverse GPCRs signaling through diverse G protein subunits. Thus, our data suggest that the inhibitory effect of probenecid is specific to the TAS2R receptors and not to downstream cellular components of G protein signaling.

### Probenecid inhibition of bitter taste receptor activation is rapid and does not involve MRP1

**Figure 4:**
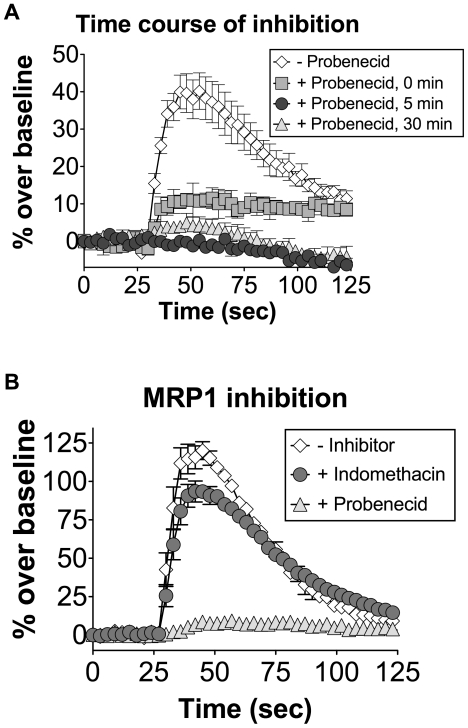
Probenecid inhibition of hTAS2R16 occurs rapidly and is not dependent on the MRP1 transporter. (A) HEK-293T cells were transiently transfected with hTAS2R16 and Gα16gust44. 22 hours post-transfection, calcium influx was measured for cells that were challenged with 3 mM salicin in the presence of 1 mM probenecid pretreatment for the indicated amount of time (0 min indicates co-injection of salicin with probenecid). hTAS2R16 was completely inactivated by 5 minutes of probenecid pretreatment. (B) HEK-293T cells were transiently transfected with hTAS2R16 and Gα16gust44 followed by challenge with 3 mM salicin in the presence or absence of the indicated compounds (1 mM, pretreatment for 60 minutes). The MRP1 transporter inhibitor indomethacin did not inhibit hTAS2R16 function. Error bars represent standard errors (n = 3).

To determine the kinetics of inhibition by probenecid, we measured the degree of inhibition of a model bitter taste receptor, hTAS2R16, following 0, 5, and 30 minute pre-incubations with probenecid (Figure 4A). Pre-incubation with probenecid for as little as 5 minutes resulted in complete inhibition of hTAS2R16 signaling. Furthermore, even simultaneous injection of probenecid with ligand gave measurable (>50%) inhibition (Figure 4A, ‘0 min’). These results demonstrate that the mechanism of action of probenecid is extremely rapid, consistent with direct inhibition of the receptors.

Probenecid is an FDA-approved inhibitor of MRP1 and other organic anion transporters [29]. To determine whether MRP1 inhibition could explain probenecid's mechanism of action, we tested the ability of indomethacin, another MRP1 inhibitor that is structurally unrelated to probenecid [28], to inhibit calcium mobilization upon hTAS2R16 activation by salicin. We found that the hTAS2R16 response to salicin was not inhibited by a 1 hr pre-incubation with 1 mM indomethacin (Figure 4B). A similar lack of inhibition using indomethacin was observed for the hTAS2R38 response to PTC (data not shown). Consistent with these results, treatment with 1 mM chloroquine, another inhibitor of MRP1, also failed to inhibit hTAS2R38 function (data not shown). The observation that hTAS2R16 and hTAS2R38 are capable of signaling in the presence of other MRP1 transporter inhibitors suggests that probenecid's mechanism of action does not occur through MRP1 or related channels.

### Pharmacology of probenecid

**Figure 5:**
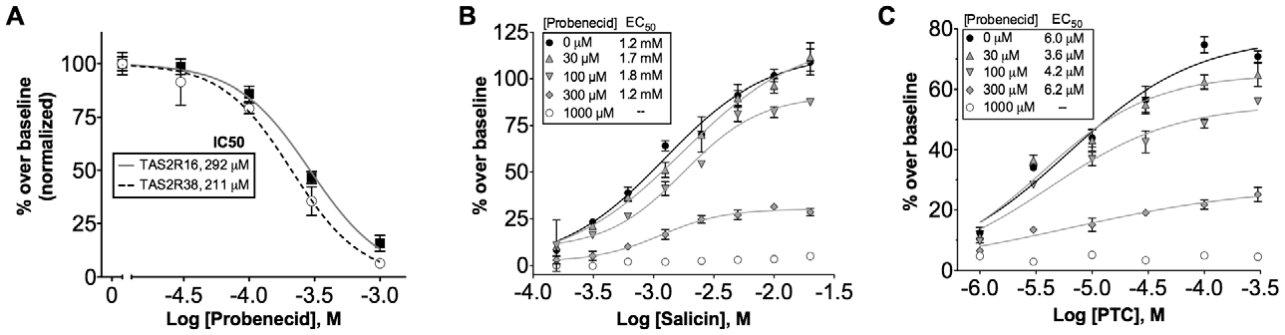
Pharmacological mechanism of action of probenecid inhibition. (A) HEK-293T cells were transiently transfected with Gα16gust44 and hTAS2R16 or hTAS2R38. 22 hours post-transfection, cells were pre-treated with increasing amounts of probenecid for 1 hour followed by challenge with 3 mM salicin or 300 µM PTC. (B) HEK-293T cells were transiently transfected with Gα16gust44 and hTAS2R16, pre-treated with increasing amounts of probenecid for 1 hour, and then challenged with different concentrations of salicin. Error bars represent standard errors (n = 4). (C) HEK-293T cells were transiently transfected with Gα16gust44 and hTAS2R38, pre-treated with increasing amounts of probenecid for 1 hour, and then challenged with different concentrations of PTC. Error bars represent standard errors (n = 4).

To assess the potency of probenecid, we measured probenecid dose responses against hTAS2R16 (in the presence of 3 mM salicin) and hTAS2R38 (in the presence of 300 µM PTC) and calculated IC50 values of 292 µM and 211 µM respectively (Figure 5A). To further characterize the mechanism of inhibition by probenecid, we generated dose-response curves for salicin-induced activation of hTAS2R16 in the presence of increasing concentrations of probenecid. In the absence of probenecid, the EC50 value of salicin was approximately 1.2 mM (Figure 5B), close to its reported value [25]. Upon the addition of increasing concentrations of probenecid, we observe a dose-dependent decrease in the maximum signal with little change in salicin EC50 values (Figure 5B), a profile typical of non-competitive allosteric inhibitors, which bind to a receptor site distinct from the orthosteric ligand binding site [35]. A similar inhibition profile was also observed for dose response curves of PTC-induced activation of hTAS2R38 (Figure 5C).

**Figure 6:**
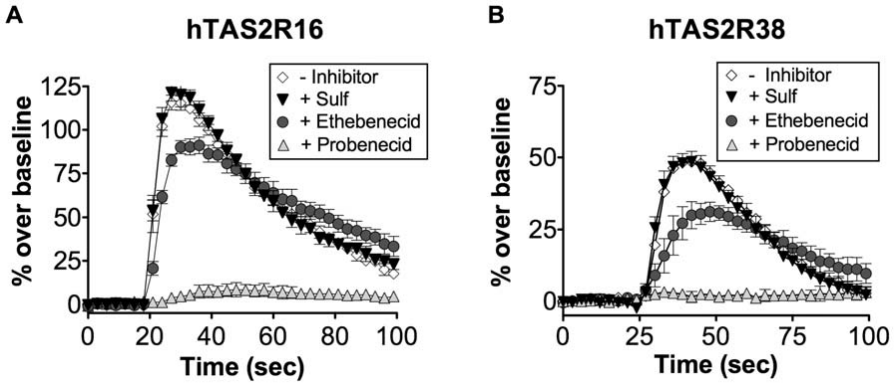
Differential effect of probenecid analogs on the activation of hTAS2R16 and hTAS2R38 receptors. HEK-293T cells were transiently transfected with Gα16gust44 and the indicated TAS2R receptor. 22 hours post-transfection, calcium influx was measured after challenge with (A) 3 mM salicin or (B) 100 µM PTC in the presence or absence of the indicated compounds (1 mM, pretreatment for 60 minutes). Error bars represent standard deviations (n = 6 for hTAS2R16; n = 12 for hTAS2R38).

To identify structural elements of the probenecid molecule that contribute to TAS2R inhibition, we tested two probenecid analogs: 4-sulfamoylbenzoic acid, comprising the core scaffold of probenecid, and ethebenecid, containing ethyl groups rather than propyl groups on the sulfamoyl group (Figure 2). Treatment with 1 mM 4-sulfamoylbenzoic acid demonstrated no inhibitory effect on either hTAS2R16 or hTAS2R38 function (Figure 6). However, treatment with 1 mM ethebenecid showed very small, but consistent, inhibition of both hTAS2R16 and hTAS2R38, suggesting that the inhibition of TAS2R function is due to specific structural moieties of probenecid. The inability of 4-sulfamoylbenzoic acid and the reduced ability of ethebenecid to inhibit TAS2R function suggests that the lengths of the acyl chains on the sulfamoyl moiety are critical for the inhibitory action of probenecid. This observation is consistent with hydrophobicity playing an important role in the inhibitory function of probenecid.

### Identification of hTAS2R16 residues required for probenecid inhibition

**Figure 7:**
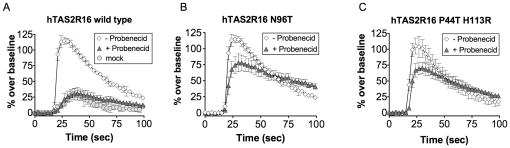
Identification of hTAS2R16 residues required for probenecid inhibition. (A) HEK-293T cells were transiently transfected with wild type hTAS2R16 and Gα16gust44. 22 hours post-transfection, calcium flux was measured for cells that were challenged with 3 mM salicin in the presence (closed triangles) or absence (open diamonds) of probenecid (1 mM; 1 hour pre-incubation). Salicin response to mock transfected (vector alone) HEK-293T cells is shown for comparison. (B, C) HEK-293T cells were similarly transfected with hTAS2R16 variants containing the mutations N96T or P44T/H113R, and challenged with 3 mM salicin in the presence or absence of probenecid (1 mM; 1 hour pre-incubation). N96T and P44T/H113R mutants showed decreased sensitivity to probenecid. A separate clone containing the single point mutant H113R was also tested to rule out this residue (Figure S1). Error bars represent standard deviations (n = 4).

To determine whether probenecid directly interacts with hTAS2R16, we screened a random mutation library of hTAS2R16 for mutations that caused a loss of inhibition by probenecid. We identified two clones containing a total of three mutations, N96T, P44T, and H113R, which were significantly insensitive to probenecid inhibition while maintaining wild-type levels of responsiveness to salicin (Fig. 7, p<0.001). Since one of the probenecid insensitive clones contained two point mutations (P44T and H113R), we also analyzed an additional clone containing only the single point mutation H113R (Figure S1). The H113R mutant demonstrates wild type calcium flux in the presence of salicin and complete inhibition in the presence of probenecid, strongly suggesting that P44T is the mutation that confers probenecid insensitivity. These data thus define two amino acid residues required for probenecid interaction and suggest a direct interaction between probenecid and hTAS2R16 that is consistent with probenecid's rapid activity. Interestingly, both P44 and N96 are predicted to be located in or near the intracellular regions of hTAS2R16 [36], [37], consistent with our dose-response profile of probenecid's mechanism of action, which suggests allosteric inhibition. Taken together, the presence of mutations in hTAS2R16 that desensitize the receptor to probenecid but not the ligand, the rapid mechanism of action of probenecid, and the dose-response profiles of hTAS2R16 to salicin in the presence of increasing amounts of probenecid suggest that probenecid interacts directly with hTAS2R16 and behaves as a negative allosteric modulator of hTAS2R16 function.

### Probenecid can modulate human bitter taste perception of the hTAS2R16 ligand salicin

**Figure 8:**
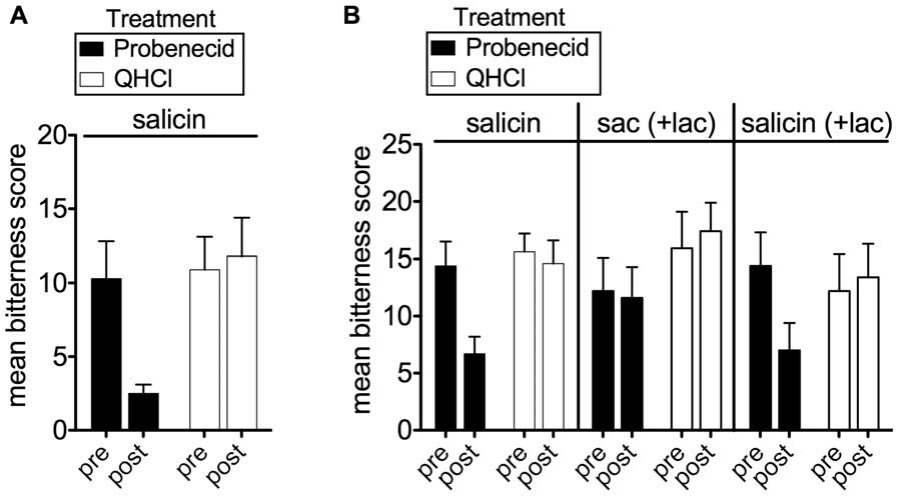
Suppression of human bitterness perception of salicin by probenecid. (A) 15 human subjects were asked to rate the bitterness intensity of 10 mM salicin before (pre) and after (post) treatment with 10 mM probenecid or control treatment with 8.1 µM quinine HCl (QHCl) on a general labeled magnitude scale (gLMS). Treatment with probenecid (black bars) significantly inhibited the perceived bitterness of salicin (p<0.05), whereas a bitter taste control treatment with QHCl had no affect (white bars). (B) 9 human subjects were asked to rate the bitterness intensity of 10 mM salicin or 250 mM saccharin before (pre) and after (post) treatment with 10 mM probenecid or control treatment with 8.1 µM quinine HCl (QHCl). Treatment with probenecid (black bars) significantly inhibited the perceived bitterness of salicin (p<0.05), whereas a control treatment with QHCl had no affect (white bars). Probenecid failed to inhibit the bitterness of saccharin (in the presence of the sweet taste inhibitor lactisole (lac) to enable subjects to focus exclusively on the bitterness of saccharin). The inhibitory effect of probenecid on salicin was also observed in the presence of lactisole, demonstrating that lactisole did not interfere with probenecid's inhibition of perceived bitterness. At the concentrations used, salicin and saccharin did not differ in their overall perceived bitterness. Error bars represent standard errors (SE).

Because multiple taste receptors, receptor alleles, and signaling pathways are involved in human taste perception, taste receptor function in cellular assays and taste perception in humans often do not correlate [38]. To determine whether hTAS2R16 receptor inhibition would translate into inhibition of bitter taste perception in humans, we assessed whether probenecid could attenuate the perceived bitterness intensity of the hTAS2R16 ligand salicin *in vivo*. Salicin is an appropriate stimulus to confirm perceptual efficacy because it interacts principally with the receptor hTAS2R16, which has relatively few receptor polymorphisms across subjects [24], [25]. Fifteen tasters were asked to rate the bitterness of a solution of 10 mM salicin on a 96 point general labeled magnitude scale (gLMS) that ranges from “Barely Detectable” to “Strongest Imaginable” [39], [40]. Tasters were then asked to rate the bitterness of salicin after rinsing with either 10 mM probenecid or, as a control bitter taste treatment, 8.1 µM quinine HCl (matched to approximate the weak bitter taste of the probenecid treatment to control for taste cross-adaptation effects). Pre-treatment with probenecid led to a significant reduction in the ability of subjects to perceive the bitter taste of salicin (Figure 8A, p<0.05), which mirrored the effect of probenecid in our cellular assay. Pre-treatment with the control solution, quinine, which is not known to interact with hTAS2R16 [41], did not inhibit the bitter taste of salicin. Importantly, 10 mM salicin evokes a moderate level of bitterness as measured by the gLMS and is a 10-fold greater concentration than the EC50 seen in human perceptual studies [25]. Thus, the reduction of salicin bitterness by probenecid is both statistically significant and perceptually robust.

We next tested for the ability of probenecid to inhibit the perceived bitterness of saccharin, a well-characterized hTAS2R31 ligand [23], [26]. Nine tasters were asked to rate the bitterness of a 250 mM saccharin solution (approximating moderate bitterness intensity levels) before and after treatment with probenecid. Because saccharin is a sweetener, we included the sweet taste inhibitor lactisole in the solutions (to enable our subjects to focus exclusively on the bitterness of saccharin). Probenecid treatment did not inhibit the perceived bitterness of saccharin, consistent with the results of our cellular assays, and the inhibitory effect of probenecid on salicin was observed again both with and without the addition of lactisole (Figure 8B). Taken together, our results demonstrate that probenecid significantly inhibits human bitter taste perception of a hTAS2R16 ligand, consistent with its mechanism of action on the hTAS2R16 receptor *in vitro*.

## Discussion

The human family of TAS2Rs is comprised of at least 25 GPCRs that are highly divergent in sequence, sharing about 30–70% amino acid homology [18], which is reflected in the ability of TAS2Rs to recognize a diverse variety of chemical moieties. Despite this diversity, only a single inhibitor of these GPCRs has been described to date [27]. Here we present evidence that an FDA-approved therapeutic is an allosteric inhibitor of a subset of human TAS2R receptors. The inhibitory properties of probenecid were unexpected since probenecid is commonly used to improve the cellular uptake of fluorescent dyes into cells to *increase* the sensitivity of GPCR calcium flux assays [31]. Our results show that probenecid can selectively inhibit the function of the bitter taste receptors hTAS2R16, hTAS2R38, and hTAS2R43 *in vitro*, while leaving intact the function of other bitter taste receptors and GPCRs, including hTAS2R31, CXCR4, CCR5, and βAR. Interestingly, the inhibition of multiple bitter taste receptors was also observed for GIV3727, a recently described hTAS2R antagonist [27]. Both probenecid and GIV3727 inhibit hTAS2R43 but only weakly inhibit hTAS2R31 (if at all), while each inhibitor has additional activity on a non-overlapping subset of receptors. The ability of both compounds to inhibit subsets of hTAS2Rs suggests that at least two different structural motifs may exist within each of these subsets of hTAS2R receptors. A better understanding of the structures of the TAS2Rs may reveal some of these common structural motifs.

Although the interaction of probenecid with a TAS2R receptor cannot be directly measured by binding or competition assays because bitter taste ligands bind too weakly (high µM to mM EC50s), our work provides several lines of evidence that the mechanism of action for probenecid inhibition occurs by direct binding to the hTAS2R16 receptor. First, analysis of hTAS2R16 point mutations define amino acid residues involved in probenecid binding or signaling that result in decreased sensitivity to probenecid while maintaining normal responses to the ligand salicin. Second, mechanism of action studies for probenecid against the hTAS2R16 receptor demonstrate rapid kinetics for complete inhibition (within 5 minutes of probenecid treatment) and near-instantaneous action for partial (>50%) inhibition, consistent with an effect on an upstream signaling component, such as the receptor itself. The effect of probenecid is also observed in the presence of inhibitors against MRP transporters (reported IC50, 100–150 µM) [42], which are responsible for probenecid's ability to increase fluorescent dye uptake. Probenecid is also known to inhibit other proteins such as the pannexin1 hemichannels in taste bud cells (IC50, 150 µM) [43], [44], but it is unlikely that inhibition of such proteins would effect GPCR signaling or explain the structural (point mutation) and mechanism of action (rapid inhibition) studies here.

The identification of point mutations at residues 44 (P44T) and 96 (N96T) of hTAS2R16 that significantly suppress the ability of probenecid to inhibit salicin activity help to define probenecid's mechanism of action on the receptor. Both mutations affect probenecid activity without affecting salicin activity, suggesting an allosteric mechanism with distinct sites on the receptor for salicin and probenecid. This is in contrast to GIV3727, where mutations in hTAS2R43 and hTAS2R46 that confer resistance to inhibition alter both the specificity and activity of agonist compounds, suggesting an overlapping binding site [27], [45]. Nevertheless, P44 and N96 are likely to constitute only part of the probenecid interaction site, as these residues are not completely conserved between the hTAS2Rs that are sensitive to probenecid (i.e. hTAS2R16, hTAS2R38, and hTAS2R43 do not all contain P44 and N96 equivalents).

As suggested by structural studies of class A GPCRs [46] and computational studies of TAS2Rs [27], [47], [48], [49], [50], the binding site for bitter receptor ligands would be expected to be in the transmembrane region of the receptor, with the site open to the extracellular portion of the receptor. Based on structure predictions, P44 and N96 are located in the first intracellular loop and the C-terminal half of the third transmembrane domain, respectively [36], [37]. Previous studies have implicated N172, located in the second extracellular loop of hTAS2R16, in the activity of diverse agonists [24]. More recently, salicin ligand docking studies and mutational analysis of hTAS2R16 demonstrate the presence of at least 7 residues in TM3, TM5, and TM6 (distinct from P44 and N96) involved in ligand recognition for hTAS2R16, with all residues located towards the extracellular face of the receptor [47]. The disparate locations of these residues and P44/N96, as well as the equivalence of the salicin response between WT and P44T/N96T, is suggestive of distinct binding sites for salicin and probenecid and point to an allosteric mechanism of action for probenecid.

The intracellular location of the probenecid binding site suggests that probenecid may potentially act by uncoupling G proteins from the receptor. If so, probenecid may be a useful reagent for understanding signal transduction for TAS2R receptors. Additional mutational analysis to further define the binding sites for probenecid and salicin on hTAS2R16 will be important for a complete understanding of the molecular mechanism of probenecid inhibition and may provide insight for the rational design of effective bitter blockers.

It is interesting to note that well-known polymorphisms in several TAS2Rs are found in the first intracellular loop, near the location of the P44 mutation in hTAS2R16 that confers probenecid resistance. For example, P49 of hTAS2R38 is located in the first intracellular loop and is part of the well-known PAV (taster) haplotype that confers sensitivity of individuals to PTC [21]. Polymorphisms of a comparable residue, W35, in the receptors hTAS2R43 and hTAS2R31 significantly modulate the activity of the receptors with their respective ligands [23]. The effect of mutations in the first intracellular loop of hTAS2R38, hTAS2R43, and hTAS2R31 highlight the role of this domain as a conserved modulator of TAS2R function.

Our studies using probenecid analogs suggest that inhibitor hydrophobicity is important for the pharmacological activity of probenecid. In particular, the propyl groups of probenecid may provide a better fit for the size and/or hydrophobicity of the putative probenecid-binding site on hTAS2R16. The ability of probenecid to cross the plasma membrane [51] due to its negative charge at physiological pH and the hydrophobic character of probenecid's di-*n*-propyl groups, is consistent with the binding of probenecid to a site on the intracellular face of the receptor, such as one that blocks binding of a G protein to the receptor.

Finally, the ability of probenecid to completely inhibit the cellular response to salicin *in vitro* provides a mechanistic explanation for its ability to inhibit human bitter taste perception of salicin *in vivo*. Future perceptual testing with a variety of diverse bitter compounds will help to determine whether the inhibitory effect of probenecid *in vivo* includes additional TAS2Rs.

## Methods

### Reagents

Salicin, probenecid, 6-n-propylthiouracil (PROP), 4-sulfamoylbenzoic acid, N,N-diethyl-4-sulfamoylbenzoic acid (ethebenecid), chloroquine diphosphate, saccharin sodium salt hydrate, aloin, quinine HCl, indomethacin, and isoproterenol were purchased from Sigma (St. Louis, MO). Probenecid (Sigma P-8761) was dissolved at 500 mM in 1 N NaOH and titrated to pH 7.0. Phenyl-β-D-glucoside was purchased from TCI (Boston, MA). SDF-1α and RANTES were purchased from Peprotech (Rocky Hill, NJ), and phenylthiourea (PTC) was purchased from Alfa Aesar (Ward Hill, MA).

### Plasmids

hTAS2R16 (N172/H222 variant) [24], hTAS2R38 (PAV) [21], hTAS2R43 (W35/H212) and hTAS2R31 (W35/M162/V227/I240) [23] were cloned by PCR directly from genomic DNA isolated from HEK-293T cells (DNeasy blood and tissue kit, Qiagen) and TOPO cloned into pcDNA3.1D-V5His (Invitrogen) and pCAGGS vectors. The first 45 amino acids of the rat somatostatin type 3 receptor, used for cell-surface targeting [25], were generated by assembly PCR, and fusion proteins with bitter taste receptors were generated by overlap-extension PCR. Point mutations of hTAS2R16 were generated by PCR and selected from a larger mutation library of hTAS2R16 (Diversify Mutagenesis kit, Clontech) [52]. Rat gustducin DNA was a kind gift of Liquan Huang (Monell Chemical Senses Center). A G_α16_ chimera containing the last 44 amino acids of rat gustducin (Gα16gust44) was generated by overlap PCR. All constructs were sequence verified.

### Calcium flux assay

HEK-293T cells were transfected with hTAS2R expression constructs using Lipofectamine 2000 (Invitrogen) in poly-lysine coated, black 384-well plates with clear bottoms (Costar) and incubated for 22 hours at 37°C. Growth media was removed and cells were washed twice with HBSS containing 20 mM HEPES, then loaded with a calcium indicator dye in HBSS containing 20 mM HEPES (Calcium 4 Assay kit, Molecular Devices) with or without 1 mM probenecid. Cells were incubated at 37°C for 1 hour in the presence of both dye and probenecid, then moved to a Flexstation II-384 (Molecular Devices) set for 32°C. After a 15-minute temperature equilibration (without washout), indicated compounds were injected (at t = ∼25 seconds) and fluorescence was measured for 100 to 180 seconds, reading every 3 seconds. Data sets were analyzed and represented as % over baseline signal using Prism 5.0 software (GraphPad Software, Inc). For Schild plots, replicates of raw calcium flux values were expressed as % over baseline signal. The mean value at 36 seconds (corresponding to the maximum flux signal) for each concentration of TAS2R ligand in the presence of the indicated concentration of probenecid was plotted against the log of ligand concentration. Data points were fit using non-linear regression in GraphPad Prism.

### Human perceptual testing

Subjects for perceptual studies were recruited and tested with a protocol approved by the Office of Regulatory Affairs at the University of Pennsylvania. Written consent was obtained on a Regulatory Affairs-approved consent form. For the saccharin stimulus and a parallel salicin stimulus, subjects were given 2.3 mM lactisole in mixture with the stimuli, to inhibit the sweet taste of saccharin. Subjects were presented with the stimuli, either 30 ml of 10 mM salicin, 250 mM saccharin plus 2.3 mM lactisole, or 10 mM salicin plus 2.3 mM lactisole in 40 ml medicine cups (Baxter), and were asked to rate the bitterness intensity on a general labeled magnitude scale (gLMS) that ranged from “Barely Detectable” to “Strongest Imaginable” along a computerized vertical 96 mm scale with magnitude labels spaced semi-logarithmically. Subjects immersed the tongue (anterior 2 cm) into the stimulus solution for five seconds while sealing their lips around their tongue to control the area of stimulation. They were then given a series of five cups containing 10 ml of either 10 mM probenecid (dissolved in 1 N NaOH and pH adjusted to 7.4 using 1 M HCl) or a control stimulus (8.1 µM quinine HCl) and asked to rinse with each cup for two minutes and expectorate after each rinse. After rinsing, subjects were again presented with the same stimulus tasted before treatment and asked to rate the bitterness intensity from the anterior tongue. All stimuli were tested blindly with both rinses in a 2×2 design. 8.1 µM quinine HCl was selected as the control rinse to match the bitterness intensity of 10 mM probenecid based on pilot testing with ten subjects to control for bitter taste cross adaptation effects of the inhibitor.

## Supporting Information

Figure S1 **Analysis of hTAS2R16 H113R single mutant for probenecid sensitivity.** HEK-293T cells were transfected with hTAS2R16 variant H113R followed by challenge with 3 mM salicin in the presence or absence of probenecid (1 mM; 1 hour pre-incubation). The H113R mutant demonstrated wild type levels of sensitivity to probenecid and salicin. The light gray trace represents inhibition of wild type hTAS2R16 in this experiment and is shown for comparison.
(TIFF)
