# Supplementary figures and images for: Probenecid Inhibits the Human Bitter Taste Receptor TAS2R16 and Suppresses Bitter Perception of Salicin
Source: PLoS One. 2011 May 24;6(5):e20123. doi: 10.1371/journal.pone.0020123 (PMC3101243; doi:10.1371/journal.pone.0020123)

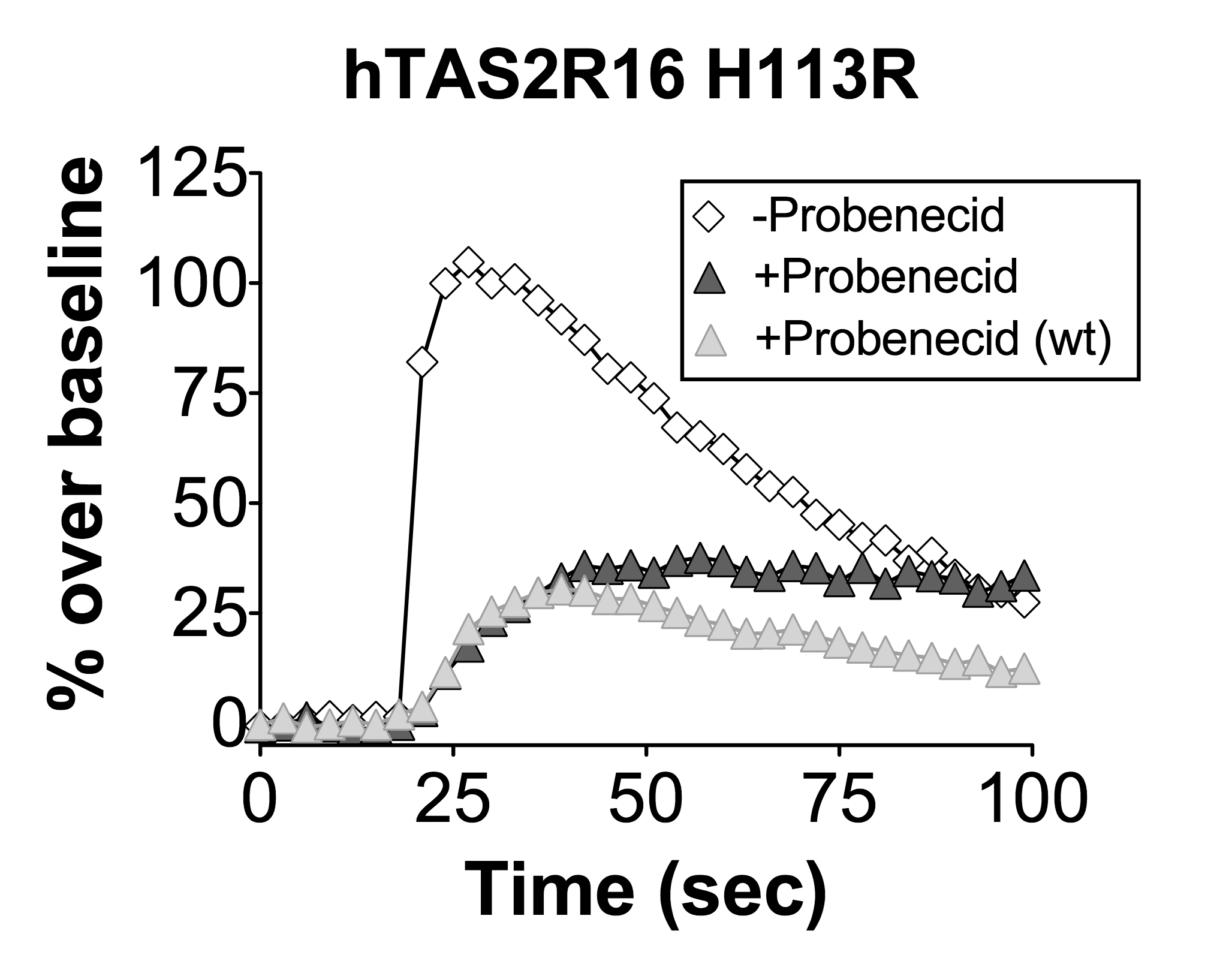

Supplement: Figure S1 — Analysis of hTAS2R16 H113R single mutant for probenecid sensitivity. HEK-293T cells were transfected with hTAS2R16 variant H113R followed by challenge with 3 mM salicin in the presence or absence of probenecid (1 mM; 1 hour pre-incubation). The H113R mutant demonstrated wild type levels of sensitivity to probenecid and salicin. The light gray trace represents inhibition of wild type hTAS2R16 in this experiment and is shown for comparison. (TIFF) [file pone.0020123.s001.tiff]
